# Effect of Greenshell^TM^ mussel on osteoarthritis biomarkers and inflammation in healthy postmenopausal women: a study protocol for a randomized double-blind placebo-controlled trial

**DOI:** 10.1186/s13063-021-05473-5

**Published:** 2021-07-28

**Authors:** Maryam Abshirini, Jane Coad, Frances M. Wolber, Pamela von Hurst, Matthew R. Miller, Hong Sabrina Tian, Marlena C. Kruger

**Affiliations:** 1 School of Health Sciences, College of Health, Massey University, Private Bag 11222, Palmerston North, 4442 New Zealand; 2 School of Food and Advanced Technology, Massey University, Palmerston North, New Zealand; 3 Centre for Metabolic Health Research, Massey University, Palmerston North, New Zealand; 4 School of Food and Nutrition, Massey University, Auckland, New Zealand; 5 Cawthron Institute, Nelson, New Zealand; 6 Sanford Ltd., Auckland, New Zealand

**Keywords:** Osteoarthritis, Biomarker, Inflammation, Greenshell mussel, Green-lipped mussel

## Abstract

**Background:**

New Zealand Greenshell™ mussels (GSM; *Perna canaliculus*) have recently been shown to decrease cartilage degradation in a rat model of induced metabolic osteoarthritis (MetOA). However, this effect has not been investigated in human subjects. This study aims to determine the effect of GSM powder on biomarkers of cartilage metabolism, bone resorption, and inflammation in New Zealand healthy overweight/obese postmenopausal women who are at early stage or at high risk of OA.

**Method:**

Fifty overweight or obese (BMI 25–35 kg/m^2^) postmenopausal women (aged 55–75 years) will be recruited by advertisement. Participants will be randomized based on a double-blind randomization schedule and stratified randomization based on BMI and age distribution. The participant will be assigned with a 1:1 allocation ratio to receive 3 g/d whole meat GSM powder or placebo (sunflower seed protein) for 12 weeks. Data on socio-demographics, physical activity, and dietary intake will be collected for each subject. Cartilage turnover biomarkers [(C-telopeptide of type II collagen (CTX-II), C-propeptide of type II procollagen (CPII), Cartilage oligomeric matrix protein (COMP)], and bone resorption marker (CTX-I) will be measured in blood and urine samples. Inflammatory status (hs-CRP and cytokine panel) will be assessed and iron status will be measured. Body composition including fat mass (FM), lean mass (LM), and fat percentage will be measured using dual-energy X-ray absorptiometry (DXA). Joint pain and knee function will be assessed using a 100-mm visual analog scale (VAS) and the Knee Injury and Osteoarthritis Outcome Score (KOOS) questionnaire, respectively.

**Discussion:**

This trial will be the first to explore the effects of whole meat GSM powder on cartilage turnover, bone resorption, and inflammation biomarkers in overweight/obese postmenopausal women. The results from this trial will provide evidence on the efficacy of GSM in the prevention of OA.

**Trial registration:**

Australian New Zealand Clinical Trials Registry (ANZCTR) ACTRN12620000413921p. Registration on 27 March 2020.

## Background

Osteoarthritis (OA) is the most common type of degenerative joint disease, contributing to progressive pain and functional loss. It is estimated to affect 33.6% of the elderly over 65 years of age [1, 2]. OA affects an estimated 3.7% of the population worldwide and that equates to approximately 268 million people [3]. The public health burden of OA is increasing, in parallel with aging and obesity. OA is more prevalent in women compared to men, and the incidence is accelerated following menopause [4]. Estrogen depletion is an important risk factor for OA, possibly due to the presence of estrogen receptors (ERs) in joint tissues [5].

Menopausal transition is accompanied by weight gain which contributes to several health risks including musculoskeletal diseases [6]. An increased body mass index (BMI) is strongly related to an increased risk of knee and hip OA [7]. Excess body weight not only increases the OA risk at weight-bearing joints due to increased mechanical loading; it has been shown to increase the risk of OA in non-weight-bearing joints such as in the hands [8]. Increased adipose tissue secretes adipokines and pro-inflammatory cytokines contributing to low-grade systemic inflammation [9]. Tumor necrosis factor-alpha (TNF-α), interleukin-1 beta (IL-1β), and interleukin-6 (IL-6) play a crucial role in cartilage loss and bone resorption in the pathogenesis of OA. These cytokines induce the production of cartilage degrading enzymes and suppress the synthesis of collagen type II and other cartilage matrix components [10].

So far, no therapeutic strategies have been proven yet and conventional treatments, particularly non-steroidal anti-inflammatory drugs (NSAIDs), have been used to reduce inflammation and symptoms. Prolonged use of NSAIDs has been associated with adverse side effects such as gastric or peptic ulcers [11]. Hence, the identification of safe interventions for disease prevention and treatment of early OA has become increasingly important. Green-lipped mussels (*Perna canaliculus*) or Greenshell™ mussels (GSM) is a known commercial aqua cultured species native to New Zealand (NZ). GSM contains high amounts of long-chain omega-3 polyunsaturated fatty acids (n-3 PUFA), eicosapentaenoic acid (20:5 n-3 EPA) and docosahexaenoic (22:6 n-3 DHA), and several other bioactive compounds [12]. With potent anti-inflammatory properties, GSM oil extracts have been shown to alleviate the arthritic symptoms [13].

The chondroprotective effect of GSM has recently been reported in an experimental model of OA. In this study, serum concentration of the cartilage degradation biomarker C-terminal telopeptides of type II collagen (CTX-II) was significantly reduced after GSM powder was added to the diet of rats actively developing metabolic OA [14]. Results from this preliminary study support the potential for an intervention study supplementing human subjects with GSM powder in order to slow the development of OA.

Obesity predisposes individuals to subclinical inflammation and concurrently reduces iron absorption and systemic iron availability from cellular iron stores [15]. Obese people are at risk of developing iron deficiency, leading to escalation of the disease burden [16]. GSM contains a high concentration of both haem and non-haem iron as well as iron absorption enhancers including myofibrillar proteins, low-molecular-weight aminoglycans, and n-3 PUFA [17]. GSM enhanced the non-haem iron uptake in human intestinal epithelial cells (Caco-2) to a similar extent to that of beef [18]. For these reasons, we hypothesize that GSM could potentially improve the iron status biomarker.

To date, there has been no previous trial investigating the effect of GSM powder on OA biomarkers in human subjects. Therefore, the current clinical trial aims to investigate the effect of powder derived from New Zealand GSM on biomarkers of cartilage turnover, inflammation, body composition, and outcomes of joint pain and knee function in healthy overweight or obese postmenopausal women who are at great risk of developing OA.

## Methods

### Study design

**Fig. 1:**
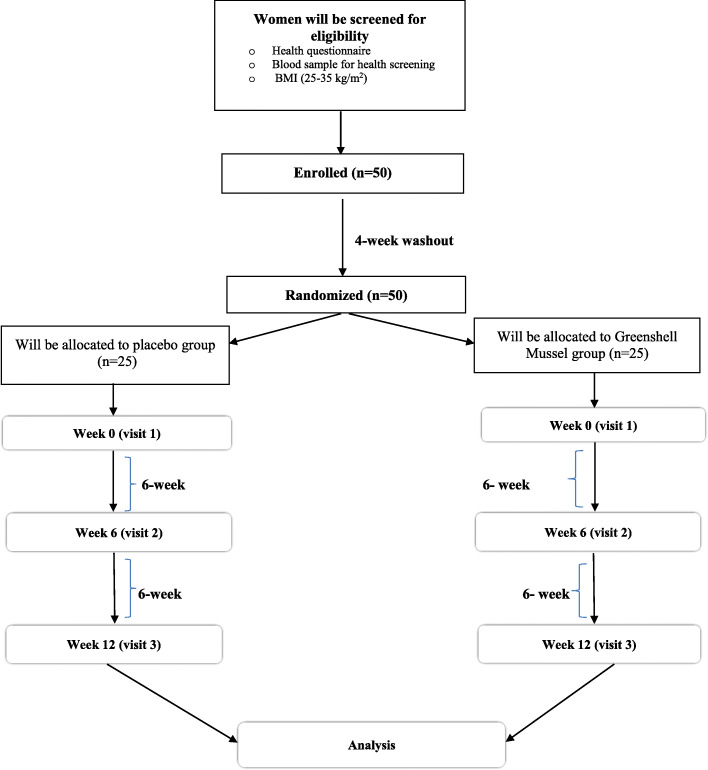
Flowchart describing the study

This is a randomized, double-blind, placebo-controlled trial. The flowchart of the study protocol is illustrated in Fig. 1. This study will be conducted at Human Nutrition Research Unit at Massey University, Palmerston North, New Zealand.

### Sample size

The sample size calculation applies to CTX-II/creatinine, CTX-II/procollagen II C-propeptide (PIICP) also referred as (CPII) CPII ratio, and cartilage oligomeric matrix protein (COMP) as the primary outcome variables. For CTX-II/ creatinine as well as CTX-II/ CPII a sample size of 24 is required to detect a 20% relative difference from baseline with 80% power. Hence, 25 subjects will be recruited for each group (GSM and placebo). For COMP as well as CPII a sample size of 7 is required to detect a 20% difference from baseline. This randomized double-blind placebo-controlled trial in parallel design recruit 55 (allowing for 10% potential dropout rate, with 27–28 sample in each arm).

### Study population

Fifty apparently healthy postmenopausal women aged 55 to 75 years will be recruited by advertisement on campus and by using a recruitment agency, Trial Facts (https://trialfacts.com/).

### Informed consent form procedure

Written informed consent will be obtained from participants before commencing data collection. The researcher provides an explanation on details of the trial, including the purpose, follow-up processes, sample collections, and other related procedures. After the eligible participant agrees to undergo the explained procedure, the research team will give the consent form to the participant to read. The participants are allowed to ask any questions regarding the consent form and trial involvement. If the participant voluntarily agrees to participate, the participant will sign the consent form. If the participant requires additional time to make more informed and voluntary decisions, the researcher will wait and allow this opportunity. Only the main researcher will be involved in obtaining the written informed consent. A copy of informed consent will be attached in the file with the study ID. The consent form is provided in Supplementary File. Currently, further analyses are not planned. Nevertheless, any future additional analysis will require institutional and ethics committee approval.

### Inclusion and exclusion criteria

For the present study, women who are at least 5 years postmenopausal (based on the natural cessation of menstruation), aged 55–75 years with no major illness, and body mass index (BMI) between 25 and 35 kg/m^2^ will be included in the study.

Patients will be excluded if (1) they have a formal diagnosis of OA or rheumatoid arthritis (RA); (2) have chronic liver or renal disease, diabetes mellitus, and atherosclerosis; (3) have a history of allergy to mussels or seafood; (4) history of joint injury or trauma; (5) smoke or have alcohol intake more than two units per day; (6) receiving supplements for joint health, multivitamins/mineral, or omega-3 regularly and unwilling to stop these 4 weeks before the beginning of the trial; (7) currently being on hormone replacement therapy or less than 6 months prior beginning the trial; and (8) continuously taking anti-inflammatory drugs or glucocorticoids or NSAIDs on a daily basis.

### Enrollment and washout period, randomization

Eligible participants will be asked to stop consumption of oily fish (salmon, sardines, pilchards, tuna, etc.), and mussels 4 weeks before the beginning of the trial until the end of the trial (from weeks - 4, to week 12). After 4 weeks of washout, a randomization list will be generated by Excel and maintained by the main investigator. Participants will be randomized based on a double-blind randomization schedule and stratified randomization will be used to match participants based on BMI (overweight: 25–29.9 kg/m^2^ and obese: 30–35 kg/m^2^) and age (55–64, 65–75 years) distribution and allocated into two groups: whole meat GSM powder (*n* = 25) and placebo groups (*n* = 25) and will be followed up for 12 weeks. The participants and researchers will be blinded to the group allocation. Table 1 shows the schedule of enrollment, interventions, and assessments.

**Table 1:** The schedule for enrollment, the interventions, and the assessments

	Study period				
	Enrollment	Allocation	Post-allocation		
Timepoint	*-t*_*2*_	*-t*_*1*_	*T*_*0*_	*T*_*6*_	*T*_*12*_
Enrollment:					
Eligibility screen	X				
Informed consent	X				
Allocation		X			
Washout period		X			
Intervention:					
Administration of GSM supplement and placebo			X	X	X
Assessments:					
Blood and urine sample collection			X	X	X
Pain assessment; VAS and KOOS			X		X
Body composition, DXA scan			X		X
Food intake; 3-day food record				X	
Compliance				X	X

### Procedure

After screening for eligibility, the informed consent form will be obtained and both groups will be invited to the Human Nutrition Research Unit at Massey University, Palmerston North three times: at baseline, week 6, and after 12 weeks. Subjects will be interviewed regarding their socio-demographic, dietary intake, and physical activity at baseline. At each visit, fasting blood and urine samples will be collected. A venous blood sample (20 ml) will be drawn after a 12-h overnight fast by a trained phlebotomist. Then blood samples will be centrifuged to obtain the serum and plasma EDTA and will be stored at − 80 °C until further biochemical analyses. All the ethical aspects pertaining to the storage of these samples have been approved by the Ethics Committee at Massey University. Body composition measurements including fat mass, lean mass, and fat percentage will be measured and analyzed using the Hologic Horizon A, Dual-energy X-ray Absorptiometry (DXA) at baseline and week 12.

Serum cartilage biomarkers CTX-II, COMP, and CPII will be assessed using commercially available sandwich enzyme immunoassay kits (BioVendor Research and Diagnostic Products, Karasek, Czech Republic). Bone marker CTX-I will be analyzed by electrochemiluminescence immunoassay using the Roche COBAS® e411 system (Roche Diagnostics, Indianapolis, IN, USA).

Urinary CTX-II will be measured using enzyme immunoassay (IDS immunodiagnostic System, Fountain Hills, Arizona, USA). Cytokine concentrations will be analyzed using BioLegend® LEGENDplex™ Multi-Analyte Flow Assay, following the manufacturer’s instructions. To determine vitamin D status at the baseline, calcidiol (25-hydroxyvitamin D [25(OH) D]) will be analyzed using isotope-dilution liquid chromatography-tandem mass spectrometry (ID-LC-MSMS).

The assays will be performed at accredited Clinical Laboratories and each specific biochemical assay analyzed will be done by the same person using the same equipment to mitigate the possible bias. We are planning to analyze vitamin D status at Canterbury Health, Christchurch, New Zealand. Serum iron, soluble transferrin receptors, transferrin, and ferritin will be measured to assess the iron status by measured at MedLab Central Palmerston North, New Zealand to provide information on iron status. Urine creatinine will also be assessed by the colorimetric method at Human Nutrition Research Unit at Massey University, Palmerston North, New Zealand. Creatinine will be used to correct the level of urinary CTX-II according to the urinary concentration.

### Intervention

Spray-dried GSM powder will be produced by Sanford Ltd (ENZAQ facility, Blenheim, New Zealand) using standard manufacturing processes. The proximate composition and microbiological load will be assessed by a commercial testing laboratory (Food Testing Laboratory of Cawthron Analytical Services; Nelson, New Zealand). The Association of Official Analytical Chemists (AOAC) methods for crude protein (AOAC 981.10), total fat (AOAC 948.15), moisture at 105 °C (AOAC 950.46) and ash (AOAC 920.153) will be used and carbohydrate content will be determined by calculation (100% − % crude protein − % total fat − % moisture − % ash). An aliquot of the total lipid extract from the GSM powder will be analyzed by gas chromatography mass spectrometry (GC-MS) according to AOAC 963.22. The placebo (sunflower seed protein powder) capsules will be purchased by Sanford Ltd from a commercial supplier. Both GSM and placebo will be encapsulated in gelatin capsules by a commercial facility (Alaron, Nelson NZ) and stored under nitrogen in the dark until use. Each capsule will contain 0.5 g of whole meat GSM powder or sunflower seed protein powder. The sunflower seed protein was selected as a placebo to provide a neutral protein source and to mimic GSM powder macronutrient composition and be as inert and non-bioactive as possible. The capsules will be completely similar in shape, size, and color (which will be dark to hide contents). Participants will receive three bottles of GSM supplement or placebo (each bottle contains 120 capsules) for 6 weeks distributed by a research at baseline. At the follow-up visit, the subjects will return their empty bottles and unused capsules to the researcher, to be counted and recorded and receive two new bottles along with their unused supplement. Participants will be required to consume 6 capsules (3 grams total) per day with meals. To allow the concealment and ability for a blinded trial, activated carbon sachets for absorbing moisture and odor in a bottle of supplement and placebo will be used; thus GSM capsule was indistinguishable from the sunflower seed protein powder used in the control group. At end of the study, the researcher and participants will be unblinded to the group allocation.

### Compliance

To assess subject’s compliance during the 12 weeks, diaries will be provided at the randomization to record the compliance to dietary and medication/supplement restriction. At weeks 6 and 12, subjects will return their unused capsules to the researcher to assess the rate of compliance. At weeks 6 and 12 consumption record diaries will be cross-checked with subjects. At the baseline and week 12, the plasma and erythrocyte membrane fatty acids will also be measured as an indicator to assess the adherence to study protocol. Participants in this trial are allowed to continue taking analgesic medications (panadol or paracetamol) as required.

### Safety

Minor gastrointestinal disturbances are expected as adverse effects based upon published literature. Information on all adverse events will be systematically collected in diaries provided by recording the history of any illness since the last visit and assessment of signs of concern at follow-up visit. Routine laboratory measurements including liver and kidney function tests, blood glucose (non-fasting), and lipid profile (triglyceride, total cholesterol, HDL-cholesterol, LDL-cholesterol) will be performed at the time of enrollment and after 12 weeks of treatment at MedLab Central Palmerston North, New Zealand by the certified phlebotomist. In case of any side effects or blood abnormalities, the person will be withdrawn and referred to their general practitioner for further examination. In case the experimental supplement is proven to be efficacious, the trial participants will be provided with a supplement post-trial.

### Monitoring and quality assurance

The quality assurance of data will be checked by a trial supervisor. M.C.K is working with a researcher for monitoring the general trial processes, completeness, and accuracy of the protocol. The trial supervisor will have access to the results of data analysis, and progress report and any adverse event will be shared with the main supervisor on a weekly basis in the form of meeting.

### Data collection and management

Participant confidentiality will be maintained through a unique ID system, and the participant identification information will not be exposed to anyone outside the trial team. All personal data will be destroyed at the end of the trial. Scientific data, filed on paper, will be shredded, and electronic data will be deleted from our computer records and databases after 10 years. For the first 5 years, it will be stored in a locked filing cupboard within a locked office. For the last 5 years, it will be stored in a secure archive where all data is stored in boxes labeled by barcode only. It is accessible by nominated staff only who require pin numbers for ID. The results of the trial will be disseminated in conference presentations, manuscripts for publication.

### Study outcomes

#### Primary outcomes

The difference in the mean of cartilage turnover markers (CPII, COMP, CTX-II) and bone resorption marker (CTX-I), measured at the end of the study in comparison with the week 6 and baseline values.

#### Secondary outcomes

The difference in the mean of inflammatory markers including hs-CRP and 13 cytokines namely IL-1β, interferon alpha-2 (IFN-α2), IFN-λ, TNF-α, monocyte chemoattractant protein-1 (MCP-1), IL-6, IL-8, IL-10, IL-12p70, IL-17A, IL-18, IL-23, and IL-33, as well as serum soluble transferrin receptor levels as an indicator of iron status measured at the end of the study in comparison with baseline values. Changes in body composition including fat mass (FM), lean mass (LM), and fat percentage, joint pain, and knee function will also be measured.

### Assessment of dietary intake

Dietary intake, a 3-day diet diary (3-DDD) over non-consecutive days (including one weekend day), will be collected one time during the study. At the first visit, an instruction on how to complete the food diary will be provided to the participants by the trained researcher. The 3-DDD will be used to collect information on participants’ food and beverage intake using household measurement tools to assist subjects in estimating the portion sizes of the food. Brand name of food products, recipes, and method of food preparation will be described. Dietary intake will be analyzed with Food works 9 professional, Xyris software.

### Assessment of physical activity

Physical activity will be assessed using the New Zealand Physical Activity Questionnaire-short form (NZPAQ-SF) [19]. The NZPAQ has previously been validated by Boon et al. Physical activity will be measured by METs-min/day which is computed by using the scoring protocol of IPAQ for continuous score [20]. MET values and formula for calculation of MET-minutes will be assessed and used as below:

- Walking MET-minutes/week at work = 3.3 × walking minutes × walking days at work
- Moderate MET-minutes/week at work = 4.0 × moderate-intensity activity minutes × moderate intensity days at work.
- Vigorous MET-minutes/week at work = 8.0 × vigorous-intensity activity minutes × vigorous
- Total Work MET-minutes/week = sum of walking + moderate + vigorous MET-minutes/week scores at work

### Assessment of joint pain and knee function

Subjects will be provided an assessment of their joint pain level using the 100 mm visual analog scale (VAS) at 6-week intervals.

Knee Injury and Osteoarthritis Outcome Score (KOOS) questionnaire will be used to measure a subject's opinion about their knee and associated problems [21]. The KOOS consists of 42 questions, which cover five domains. These include (1) pain frequency and severity during functional activities; (2) severity of knee stiffness and swelling, grinding or clicking, catching, and limitation in range of motion; (3) difficulty in performing the daily living activities; (4) difficulty experienced during sport and recreational activities; and (5) knee-related quality of life (QOL).

All items are scored on a 5-point Likert scale (0–4). The five dimensions are scored separately as the sum of all corresponding items. Scores are then converted to a 0–100 scale (percentage of total possible score obtained), where zero represents extreme knee problems and 100 represents no knee problems.

### Statistical analysis

Statistical analysis will be performed using IBM statistics software version 25 (Armonk, NY, USA). The values of variables will be presented as mean ± standard deviation. Normality tests will be assessed through Shapiro-Wilk tests carried out on each parameter before analysis. Analysis includes all the participants (intent to treat) determining the baseline characteristic. Additional analysis will be performed per protocol on participants with 80% or higher adherence to study protocol. Two-way repeated measures ANOVA will be used to determine differences within groups (pre vs. post) as a repeated factor and intervention (GSM vs. placebo) as a fixed factor, with the model adjusted for the BMI and age, physical activity, and baseline vitamin D status. Post hoc analysis with Bonferroni adjustments will be performed for multiple comparisons. Statistical significance will be set at a level of 0.05.

## Discussion

To the best of our knowledge, this is the first randomized controlled trial that will determine the effect of whole meat GSM powder on cartilage metabolism, bone resorption, and inflammation biomarkers in overweight and obese postmenopausal women who at high risk of developing OA. The current study builds on a promising pre-clinical trial [14] which confirmed the chondroprotective effect of GSM powder in an experimental model of metabolic OA by reducing the CTX-II (decreasing type II collagen degradation) without any apparent adverse effects.

The current study aims to address several gaps in the current scientific evidence for GSM interventions. Firstly, until now there have been a limited number of studies which investigated the effectiveness of whole GSM extract interventions on clinical symptoms of OA in individuals with diseased state [13, 22]. The current study will include biomarkers of cartilage turnover and inflammation to address the paucity of evidence on cartilage-protective effectiveness of GSM intervention in healthy individuals without OA. Secondly, this trial aims to target overweight and obese postmenopausal women, a population group more severely impacted by OA [23], and given the limited number of interventions in this population, this study will address this specific gap in the current evidence base.

The current study has limitations. Firstly, participants are postmenopausal women, therefore findings cannot be extrapolated to a male population. Secondly, this study aims to investigate the preventive effects of GSM against early-stage OA associated with obesity and metabolic syndrome at healthy individuals. Given the fact that stage and phenotype of OA with distinct etiology could have an impact in terms of treatment response, therefore patients with age or trauma-induced or advanced OA may not benefit from the result of this study [24].

To conclude, if the whole meat GSM intervention reduces the cartilage degradation and improves the cartilage synthesis, there is potential for the wide implementation of GSM powders as dietary supplements or functional foods, or even as a whole food to prevent or delay the onset of OA.

## Trial status

Recruitment began on 1 July 2020. Currently, recruitment is completed, and data collection is expected to be completed in September 2021. The current protocol version is 3.0, dated 27 May 2021.

## Data Availability

The data that support the findings of this study are available from the corresponding author, M.C.K upon reasonable request.
